# IGFN1_v1 is required for myoblast fusion and differentiation

**DOI:** 10.1371/journal.pone.0180217

**Published:** 2017-06-30

**Authors:** Xiang Li, Jane Baker, Tobias Cracknell, Andrew R. Haynes, Gonzalo Blanco

**Affiliations:** 1 Department of Biology, University of York, York, United Kingdom; 2 MRC Harwell Institute, Mammalian Genetics Unit, Harwell, United Kingdom; Tohoku University, JAPAN

## Abstract

*Igfn1* is a complex locus that codes for multiple splicing variants of Immunoglobulin- and Fibronectin-like domain containing proteins predominantly expressed in skeletal muscle. To reveal possible roles for *Igfn1*, we applied non-selective knock-down by shRNAs as well as specific targeting of *Igfn1* exon 13 by CRISPR/Cas9 mutagenesis in C2C12 cells. Decreased expression of *Igfn1* variants via shRNAs against the common 3’-UTR region caused a total blunting of myoblast fusion, but did not prevent expression of differentiation markers. Targeting of N-terminal domains by elimination of exon 13 via CRISPR/Cas9 mediated homologous recombination, also resulted in fusion defects as well as large multinucleated cells. Expression of IGFN1_v1 partially rescued fusion and myotube morphology in the *Igfn1* exon 13 knock-out cell line, indicating a role for this variant in myoblast fusion and differentiation. However, in vivo overexpression of IGFN1_v1 or the *Igfn1* Exon 13 CRISPR/Cas9 targeting vector did not result in significant size changes in transfected fibres.

## Introduction

*Igfn1* was identified as a protein fragment in a Yeast-two-hybrid assay using the KY protein as bait [1]. Mutations in *KY* underlie muscle disease in mouse and humans and a blunted hypertrophic response in mouse adult skeletal muscle [2][3][4]. *Igfn1* defines a genomic locus of complex transcriptional activity that produces multiple proteins predominantly in skeletal muscle and heart [5]. The largest IGFN1 protein isoform (referred to as IGFN1) contains a series of 11 fibronectin and immunoglobulin-like domains distributed in three N- and eight C-terminal domains separated by a large disordered segment. The large disordered segment is alternatively spliced in the smaller IGFN1_v1 isoform [5]. Full length cDNAs have previously been cloned that code for isoforms containing subsets of C-terminal domains only [5]. The domain composition of IGFN1 is reminiscent of other sarcomeric proteins associated with the actin cytoskeleton (e.g., myosin binding protein C, filamin C, myotilin, myopalladin or titin, see [6] for a review). These proteins are proposed to act as crosslinkers and bear an inherent flexible structure that allows them to maintain protein interactions through cycles of contraction and relaxation [7].

The function of IGFN1 is unknown. In skeletal muscle, Z-disc and nuclear localizations have been identified with antibodies that do not discriminate specific isoforms [5]. Moreover, expression of *Igfn1* has been reported to increase under different atrophy promoting conditions. Thus, transcription of *Igfn1* has a strong negative correlation with levels of myostatin signaling, the most powerful negative regulator of muscle growth [8]. Inhibition of myostatin signaling leads to muscle hypertrophy and downregulation of *Igfn1* *[9]*. Conversely, enhancement of myostatin signaling leads to muscle atrophy and dramatic upregulation of *Igfn1* expression [10]. Moreover, *Igfn1* transcripts are induced 100-fold in muscles rendered mechanically inert by denervation [11].

To reveal possible roles for *Igfn1*, we applied non-selective knock-down by shRNAs as well as specific targeting of *Igfn1* exon 13 by CRISPR/Cas9 mutagenesis in C2C12 cells. The differentiation potential of the resulting clones was evaluated. Our results suggest a role for IGFN1_v1 in myoblast fusion and myotube morphology and size when assessed in the myoblast C2C12 cell line. However, neither overexpression of IGFN1_v1 or the *Igfn1* Exon 13 CRISPR/Cas9 targeting vector in vivo resulted in significant fibre size changes, indicating that IGFN1_v1 is not sufficient to regulate fibre size in vivo.

## Results

### Selection of IGFN1 knock-down clones

**Fig 1:**
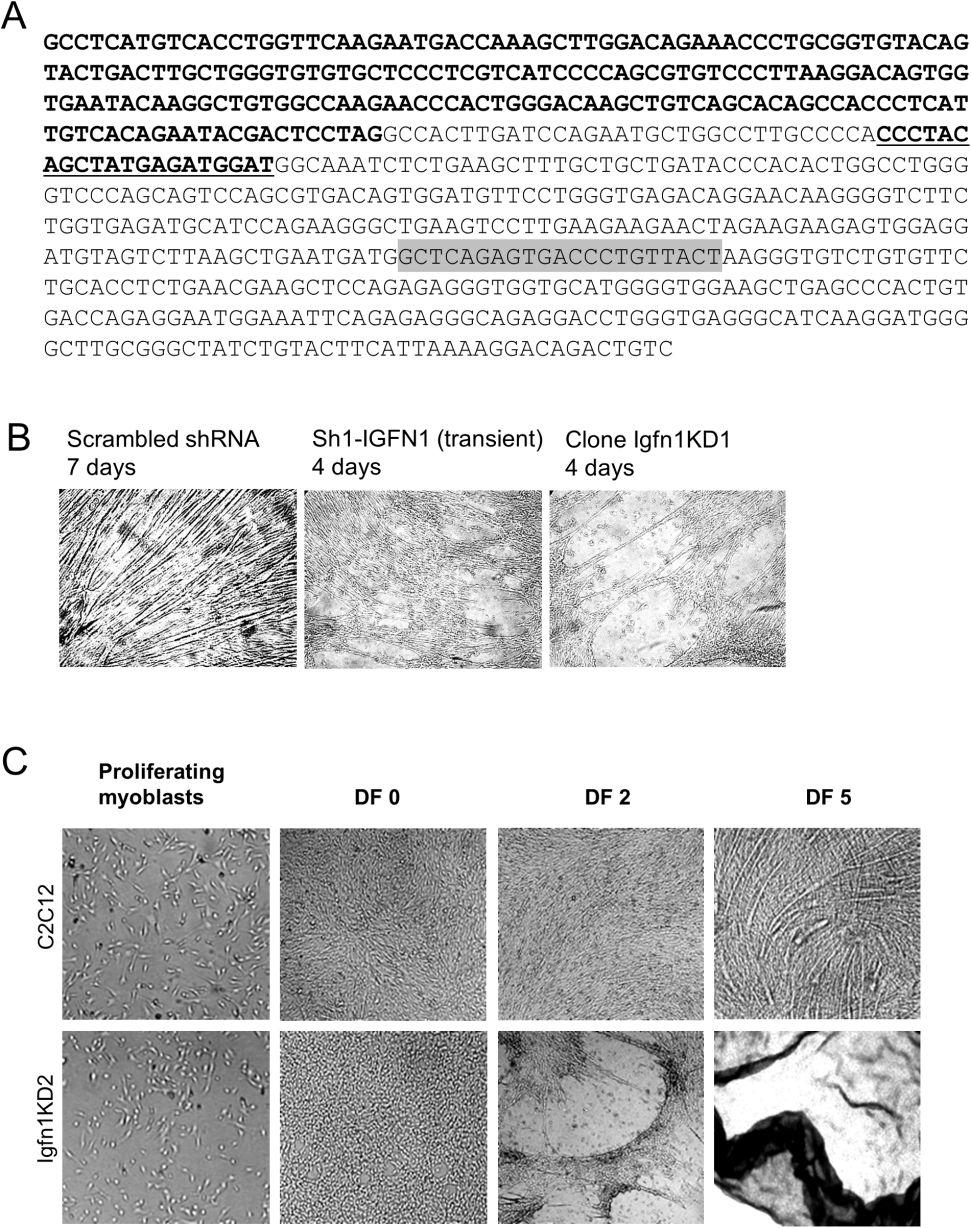
Knock-down of IGFN1 provokes cell detachment and fusion impairment. A) DNA sequence of *Igfn1* 3’UTR genomic region showing the coding sequence in bold. The sequence targeted by sh-2-IGFN1 within the 3’UTR is indicated in bold underlined. The sequence targeted by sh-1-IGFN1 is boxed in gray. B) As labelled, cultures of C2C12 cells transiently transduced with scrambled shRNA (7 days in DF medium), sh1-IGFN1 (4 days in DF medium) and selected clone Igfn1KD1 (4 days in DF medium). Note detachment of Sh1-IGFN1 transiently transduced and stably selected (Clone Igfn1KD1) cells. C) Igfn1KD2, a clone selected following transduction with sh-2-IGFN1, shows detachment upon switching to DF medium, whilst the control C2C12 cell line forms myotubes.

In order to explore an involvement of the *Igfn1* locus in myogenic differentiation a short hairpin RNA (sh-1-IGFN1) targeting the common 3’UTR identified in several variants (Fig 1A, [5]) was cloned into the pAd/BLOCK-iT™-DEST vector to produce viral particles carrying sh-1-IGFN1. C2C12 proliferating myoblasts were transduced with sh-1-IGFN1 viruses using a scrambled shRNA as control. Transduced cells with sh-1-IGFN1 proliferated at normal rates. Upon shifting to a lower serum containing medium (DF medium) to induce fusion and differentiation, sh-1-IGFN1 transduced cells detached from the plates within 4/5 days (Fig 1B). Over the 4 to 5 days in differentiation medium, these cells remained mononucleated without any evidence of cell fusion and myotube formation, while the control cell line was already forming myotubes (Fig 1B). To confirm this cellular phenotype, sh-1-IGFN1 transfected cells were clonally selected. Four selected lines showed lack of fusion and detachment after 4/5 days in differentiation medium (see clone Igfn1KD1 in Fig 1B). To confirm these results, a second commercially available lentiviral shRNA targeting a different portion of the IGFN1 3’UTR (sh-2-IGFN1, see Methods for details) was used for transient viral transductions as well as for clonal selection. Transiently transduced cells as well as two sh-2-IGFN1 selected cell lines (clones Igfn1KD2 and Igfn1KD3) failed to fuse, remaining as confluent myoblasts after several days in differentiation medium before detaching, whilst non treated C2C12 cells differentiated normally (Fig 1C).

**Fig 2:**
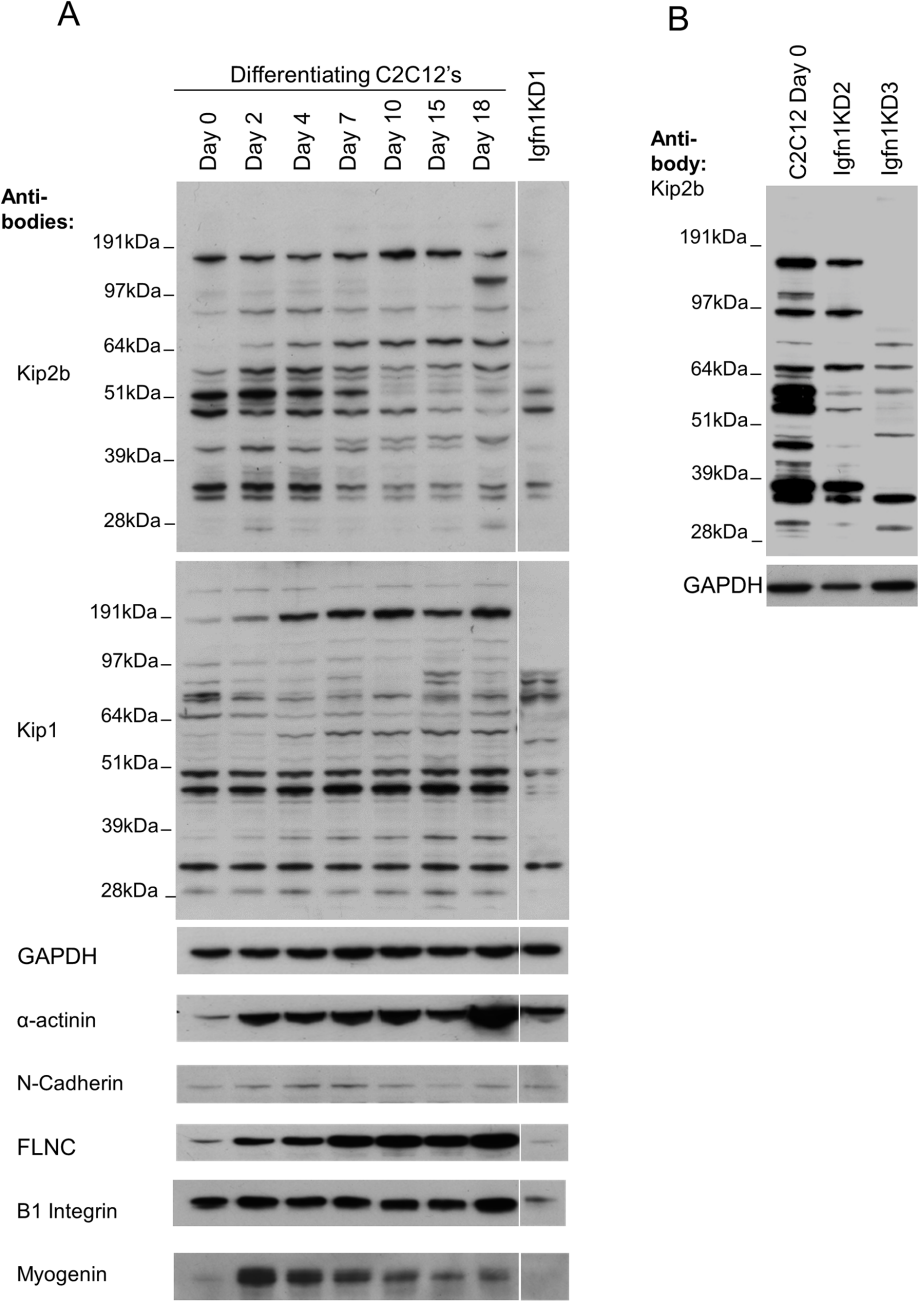
Knock-down of IGFN1 isoforms by shRNAs does not prevent expression of differentiation markers. A) Analysis of IGFN1 variants, as identified with antibodies against IGFN1 Kip2b and Kip1, and markers of differentiation throughout in vitro differentiation of C2C12 myoblasts as indicated. The indicated Igfn1KD1 lane corresponds to a stable cell line selected with sh-1-IGFN1 targeting the 3’UTR of Igfn1 collected at 4 days in DF medium. Note depletion of the largest IGFN1 protein variants. B) Two knock down clones, obtained with sh-2-IGFN1 targeting a different sequence of IGFN1 3’ UTR, showing decreased complexity of IGFN1 products compared to the C2C12 cell line, as labelled.

Down-regulation of IGFN1 isoforms in the knock-down cell lines was tested using western blots (Fig 2). We previously showed that western blots of skeletal muscle samples with anti-IGFN1 antibodies are complex, requiring more than one independent antibody to attest the identity of the bands as genuine IGFN1 products [5]. Antibodies Kip2b and Kip1 against IGFN1 have been previously shown to produce identical high molecular weight bands in western blots from skeletal muscle samples [5] and were used to profile IGFN1 expression throughout C2C12 myoblast differentiation from 0 to 18 days in DF medium (Fig 2A). When the full range of protein sizes is exposed, the profile of bands produced by these antibodies throughout differentiation appears different (Fig 2A). Nonetheless, the bands from the Igfn1KD1 cell line extracts collected at DF day 2 before detachment were lower in number and intensity compared to differentiating C2C12s (Fig 2A). Moreover, the knockdown cell lines Igfn1KD2 and Igfn1KD3, which were selected with an independent shRNA, also showed an overall decrease of putative IGFN1 products (Fig 2B).

### Characterization of Igfn1KD1

**Fig 3:**
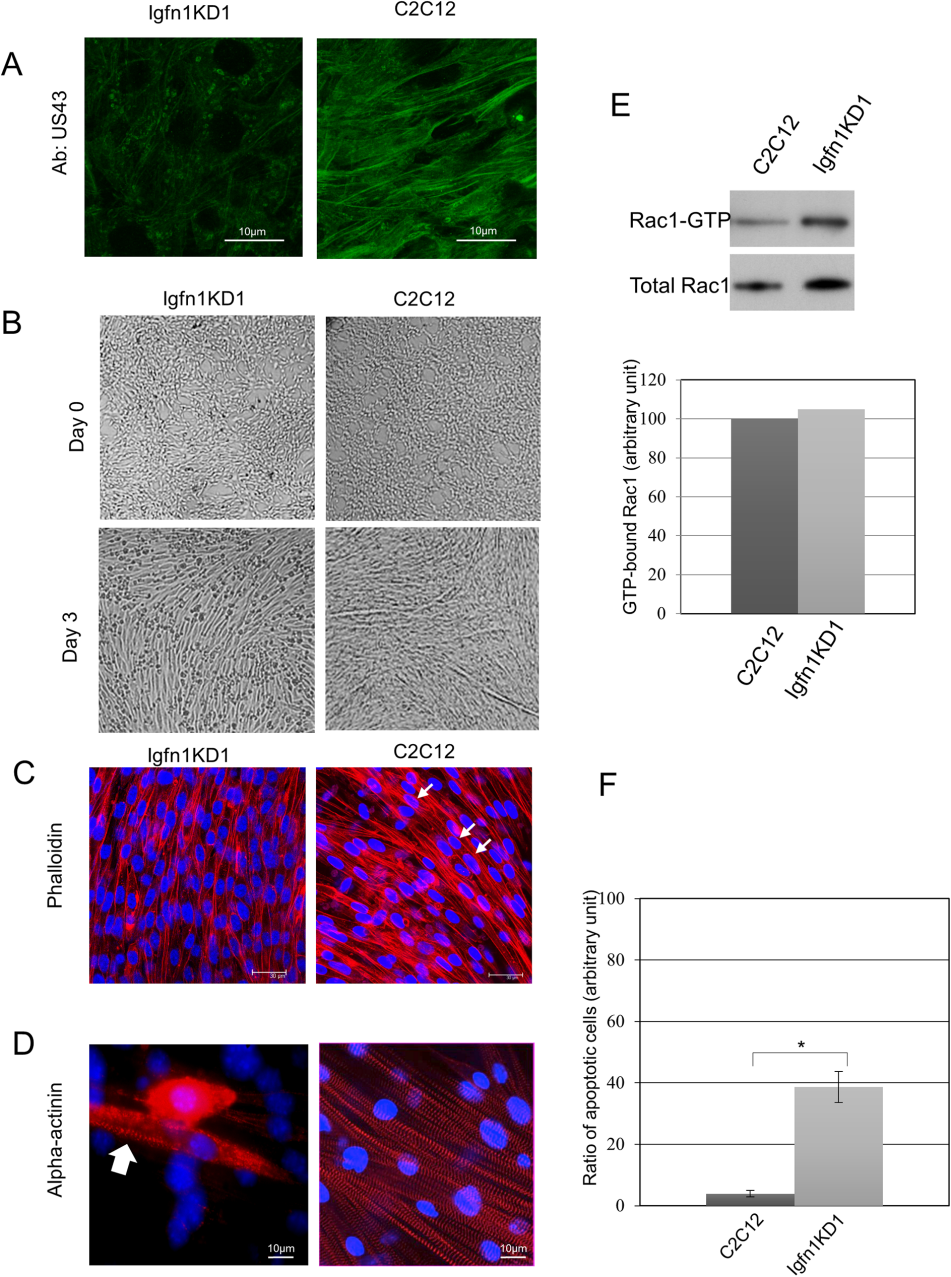
Characterization of Igfn1KD1 cell line. A) Confocal images of Igfn1KD1 and C2C12 cells obtained with Ab-US43 antibodies against IGFN1, as labelled. Note a diminished signal in the Igfn1KD1 cell line. Images were obtained with identical confocal settings. B) Igfn1KD1 fails to form myotubes on collagen coated dishes. Brightfield images of Igfn1KD1 and C2C12 at day 0 and day 3 in DF medium as labelled. C) Confocal images of Igfn1KD1 and C2C12 cells stained with phalloidin (red) at Day 2 of differentiation. White arrows point at nuclei within a single myotube identified by the uninterrupted actin filaments. Images are representative of three independent experiments and obtained with identical confocal settings. Nuclei stained with DAPI (blue). Size bar = 30μm. D) Immunofluorescence images of Igfn1KD1 and control C2C12 cells maintained for 14 days in DF medium and labelled with antibodies against alpha-actinin, as indicated. White arrow points at striations within a single Igfn1KD1 mononucleated cell that survived detachment. E) Western blots (top panels) and quantification (histogram) of relative active Rac1 levels in control and Igfn1KD1 cell lines. The histogram shows the average amount of active Rac1-GTP normalised to the total amount of Rac1 by densitometry analysis for two independent experiments. Similar levels of active Rac1 are observed in the Igfn1KD1 cell line and the control. F) Quantification analysis of apoptosis in control and Igfn1KD1 cell lines at day 4 of differentiation. The histogram shows the average quantification of number of cells undergoing apoptosis from six independent experiments, expressed as the ratio of apoptotic/non apoptotic cells (see Methods for details). (*) Differences are significant (p<0.001): t = 6.11.

We selected Igfn1KD1 for further characterizations, as this cell line appeared to have the strongest downregulation of IGFN1 isoforms (Fig 2A). Confocal images obtained using Ab-US43 antibodies against IGFN1 showed a diminished signal on Igfn1KD1 proliferating cells (Fig 3A), supporting that expression of IGFN1 isoforms has been reduced in this cell line. We tested whether laminin, gelatin and collagen coating would improve attachment of the knock-down cells after switching to DF medium. Only collagen coated dishes appeared to improve and extend attachment over a week. On collagen coated dishes, myotubes could be observed on the control C2C12 after three days in DF medium, whilst Igfn1KD1 cells remained confluent but unfused (Fig 3B). Phalloidin staining confirmed that Igfn1KD1 cells remained mononucleated, in contrast to the presence of a number of multinucleated cells in the control (Fig 3C). Phalloidin staining was less intense in the Igfn1KD1 cell line using the same confocal settings, suggesting lower levels of actin reorganization. Furthermore, the Igfn1KD1 myoblasts also appeared to have a slightly reduced bipolar shape in comparison to the control. To determine whether differentiation was also inhibited in the knock-down cell line we looked at markers of adhesion and differentiation and, for reference, these markers were profiled throughout C2C12 differentiation (Fig 2A). Cell adhesion markers cadherin and beta-integrin were detected in Igfn1KD1 cell line, although beta-integrin at lower levels than control. Markers of early (myogenin) and late differentiation (FilaminC and alpha-actinin) also showed a difference in expression. Myogenin is a marker of terminal myogenic differentiation [12] that in vitro increases expression at the early differentiation stage when myoblasts fuse and decrease expression as the myotubes mature (Fig 2A). Myogenin was barely detectable in the knock-down cell line (Fig 2A). Despite the low levels of myogenin, the Igfn1KD1 cells expressed markers of terminal differentiation alpha-actinin and filamin C [13] (Fig 2A). This was somewhat unexpected from cells that remained mononucleated since alpha-actinin is a component of the sarcomeric z-disc. However, immunoflorescence with alpha-actinin antibodies of cells that survived detachment on collagen coated dishes showed clear striations reminiscent of a sarcomeric pattern (Fig 3D). Thus, in the Igfn1KD1 cell line, terminal differentiation can still occur, but striations are reduced and look abnormal, consistent with the reduced FLNC and alpha-actinin expressions shown in Fig 2. Altogether, the results suggest a role for IGFN1 variants in myoblast fusion independent of terminal differentiation.

The apparent lower actin remodelling exposed by phalloidin (Fig 3D) prompted us to look into the activation levels of RAC1, a small GTPase involved in actin dynamics and myoblast fusion [14]. Rac1 has been implicated in myoblast fusion in *Drosophila* [15][16] [17] and in mouse C2C12 myoblasts [17,18]. In these cells, activation of Rac1 distinctively peaks at day 2 in DF medium [18], at the onset of myoblast fusion. We therefore measured the levels of active Rac1 in the Igfn1KD1 and control cell lines at day 2 of differentiation. The results indicate that activation levels or Rac1 are not affected in Igfn11KD1 cells (Fig 3E). On the other hand, apoptosis was significantly increased in the Igfn1KD1 cell line (Fig 3F).

**Fig 4:**
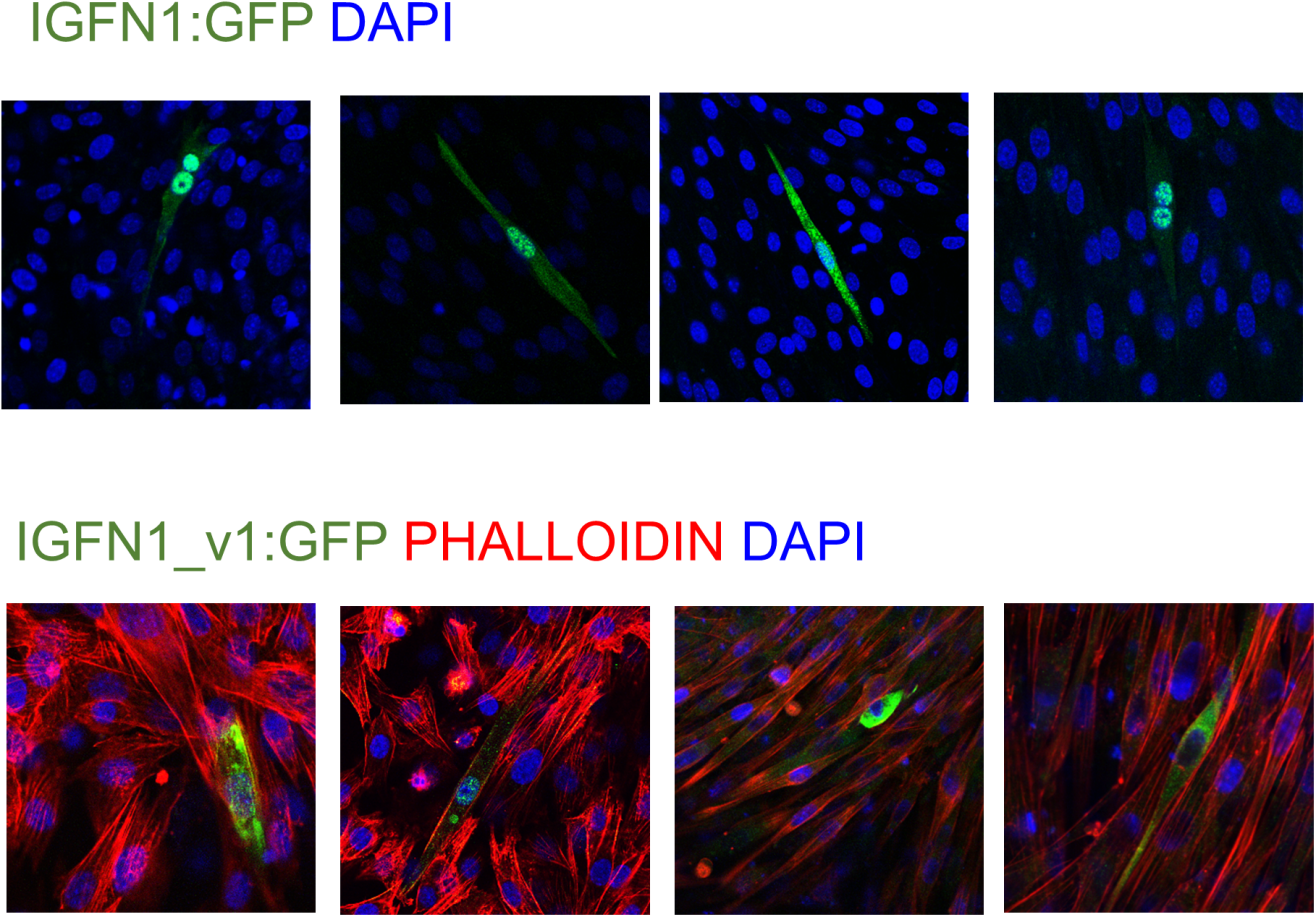
Expression of recombinant IGFN1:GFP or IGFN1_v1:GFP in the Igfn1KD cell lines does not result in rescue of myoblast fusion. Confocal images show representative examples of transfected cells in cultures maintained in DF medium for a week. Transfected cells contained one or two nuclei. Phalloidin staining (red) was used on cells transfected with IGFN1_v1:GFP only.

The fusion defect and molecular phenotypes exposed by the analyses above cannot be attributed to the loss of any specific isoform, given the apparent variety of IGFN1 isoforms showing reduced expression on western blots. Attempts to rescue the fusion defect in the Igfn1KD1 cell line using vectors coding for isoforms containing the full set of globular domains (full length IGFN1 and IGFN1_v1) failed. Lipofectamine or nucleofection based transfections with these large recombinant cDNAs were low (<1%) compared to transfections with the GFP only control (up to 25%, data not shown), which may have prevented proper assessment of fusion. However, we imaged >30 positively transfected cells with either construct after one week in DF medium on collagen plates and none of them showed >2 nuclei (a panel of examples is shown in Fig 4, with IGFN1_v1 transfections also stained with phalloidin). Since the fusion defects in the knock-down cell line could not be proven to be caused by the absence of any specific IGFN1 isoform, we opted for generating an alternative model to reassess the role of IGFN1 in myoblast fusion and myotube differentiation.

### Deletion of Exon 13 of IGFN1 by CRISPR/Cas9 mediated homologous recombination

**Fig 5:**
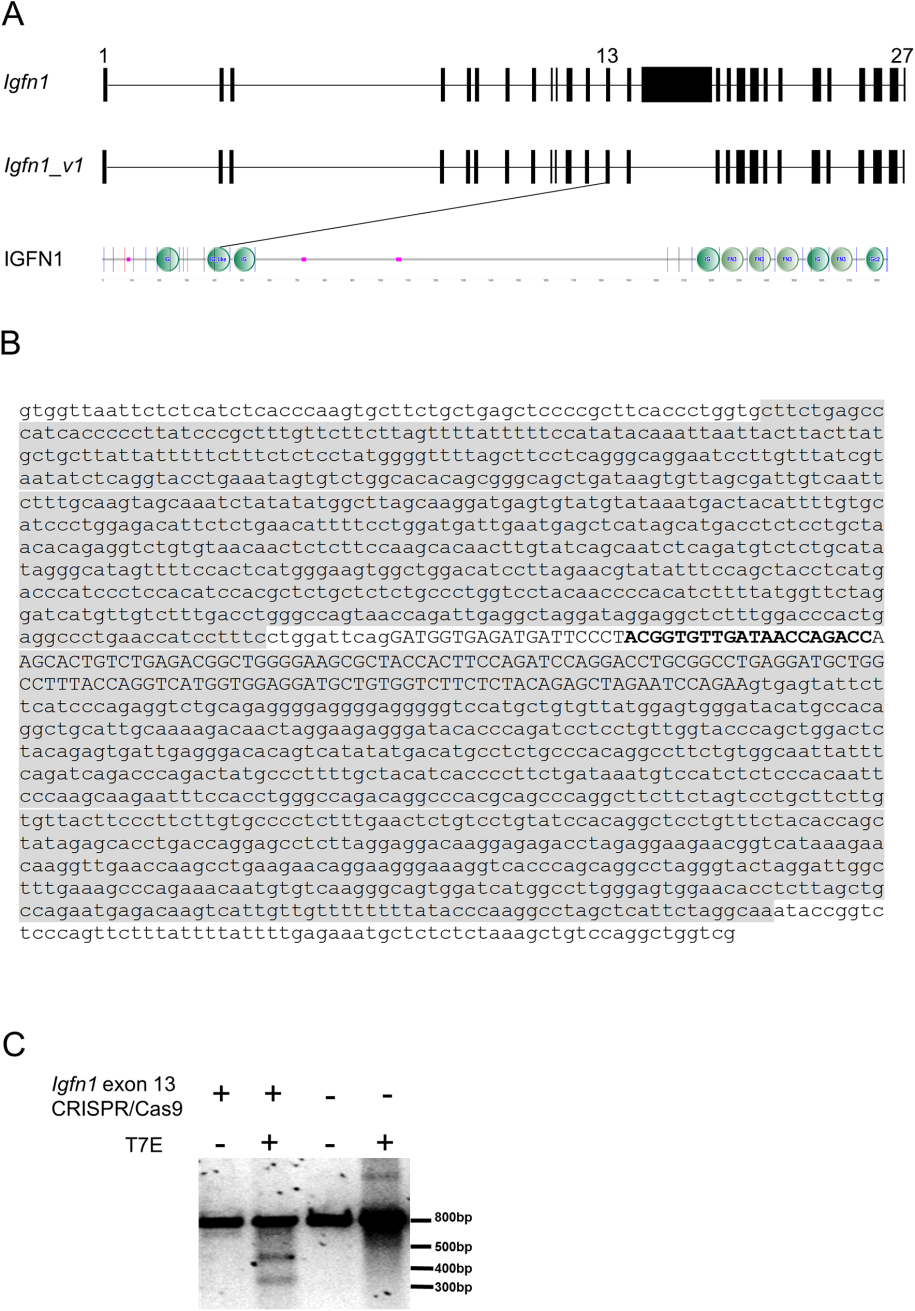
Design and validation of an *Igfn1 exon 13* CRISPR/Cas9 targeting vector. A) Genomic structure of *Igfn1* and *Igfn1_v1* and predicted domain composition of IGFN1. The first exon, exon 13 and the last exon are indicated. The SMART (http://smart.embl-heidelberg.de) predicted domain composition of full length IGFN1 is shown, with vertical lines indicating exon boundaries. Note that exon 13 codes for part of the second globular domain of IGFN1. B) Genomic sequence surrounding *Igfn1* exon 13. Exon 13 is indicated in capital letters. The CRISPR/CAS targeting sequence is indicated in bold. Left and right homologous recombination arms are boxed in grey. C) Validation of *Igfn1* exon 13 CRISPR/Cas9 targeting vector. A T7 endonuclease I digestion of the 852bp amplicon encompassing the targeted region. Note that cleavage products of the expected sizes (505bp and 347bp) are only obtained in digested (+ T7E) sample from transfected 3T3 cells (+ *Igfn1* exon 13 CRISPR/Cas9).

To ascertain a role for IGFN1 in myoblast fusion, a number of clones containing disruptive mutations were generated by CRISPR/Cas9 mutagenesis. For this, we avoided targeting the predicted first exon (Fig 5A) as it is highly likely that internal promoters and other in frame ATG codons contribute to the expression of isoforms from this locus. We also noted that exon 1 (see genomic structure in Fig 5A) is not conserved in closely related species, including rats. We chose exon 13 because it codes for the second Immunoglobulin-like domain (see Fig 5A for the predicted domain composition of full length IGFN1) and is not a multiple of 3, therefore its loss would result in a frameshift and most likely non-sense mediated decay of the resulting transcripts. To generate C2C12 derived mutant clones, a homologous recombination plasmid template that introduces puromycin and GFP as selectable and reporter markers, respectively, was used in combination with a CRISPR/Cas9 vector targeting exon 13 (Fig 5B). Targeting efficiency of the vector was tested using a mismatch cleavage assay. Genomic DNA from transfected 3T3 cells was used as template to generate an amplicon with primers flanking the targeted site. Upon T7ENI digestion, the anticipated ~505 bp and ~347 bp products were obtained only in samples from transfected 3T3 cells (Fig 5C).

**Fig 6:**
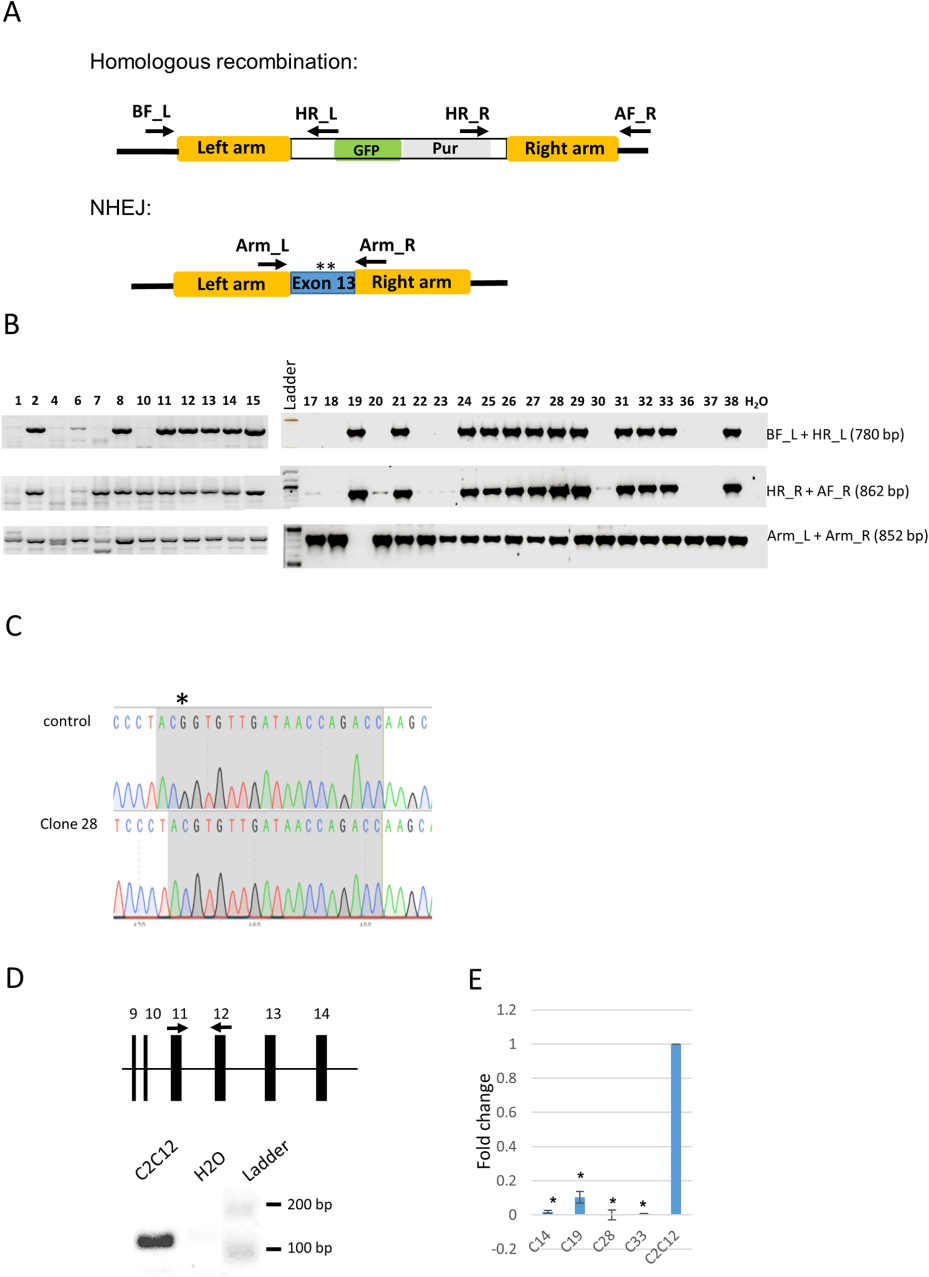
Targeting of *Igfn1 exon 13* by CRISPR/Cas9 mutagenesis. A) Schematics of the expected genomic arrangements that follows repair of double strand breaks either by homologous recombination or non-homologous end joining (NHEJ), as indicated. Homologous recombination using left (“Left arm”) and right (“Right arm”) homologous region results in replacement of exon 13 by a cassette containing GFP and puromycin reporter and selection markers, respectively. NHEJ results in random mutations inserted in exon 13, represented by asterisks. The position and labels of the screening primers are indicated. B) Screening of C2C12 derived puromycin resistant clones. PCR products of the correct size obtained with BF_L + HR_L and HR_R + AF_R indicate the presence of at least one homologous recombined allele. Non-recombined alleles produce a product of 852bp with primers Arm_L + Arm_R. Note that only clone 19 failed to produce a product for the non-recombined allele, indicating that both alleles have been recombined in this clone. C) Example of NHEJ allele (clone 28) showing a single nucleotide deletion (asterisk) aligned to the control non-targeted allele. D) Validation of qRT-PCR primers. The position of the primers on exon 11 and 12 is indicated by black arrows on the relevant segment of the *Igfn1* genomic structure (top). cDNA from C2C12 cells produced a single product of the expected size (141 bp) in a standard PCR assay, as labelled. E) qRT-PCR results from clones carrying disruptive mutations in exon 13 (C14, C19, C28, C33) and C212 control, expressed as fold change relative to the levels of C2C12, as indicated. Error bars represent the standard deviation of three technical replicates. (*) indicates significant difference (P≤0.05) to the C2C12 level.

Following transfections of C2C12 cells, a number of puromycin resistant colonies were selected and expanded. PCR amplifications using primers within the homologous recombination cassette in combination with primers on the flanking genomic regions (see position of primers in Fig 6A) identified a number of colonies showing successful homologous recombination for at least one of the two alleles (clones 2, 8, 11, 12, 13, 14, 15, 19, 21, 24, 25, 26, 27, 28, 29, 31, 32, 33 and 38, Fig 6B). The presence of a non-homologous recombined allele was determined by PCR primers within the homologous arms, which produce a 852 bp amplicon (Fig 6A and 6B). This showed that clone 19 was the only one with both alleles recombined (Fig 6B). Sequencing of the 852 bp amplicon from 18 other clones showed mutations produced by non-homologous end joining (NHEJ) in all those clones (see example in Fig 6C), altogether indicating that the targeting vector was efficiently introducing mutations at the intended site.

To test for non-sense mediated decay, clones carrying disruptive mutations in both alleles were tested by quantitative RT-PCR with primers spanning 11th and 12th exon junction, upstream of the targeted exon (Fig 6D). These primers produced a single product of the expected size (Fig 6D) on standard PCR. qRT-PCR results showed a significant reduction of transcript levels relative to the control C2C12 in all tested clones (Fig 6E).

### IGFN1 KO clones display fusion and differentiation defects

**Fig 7:**
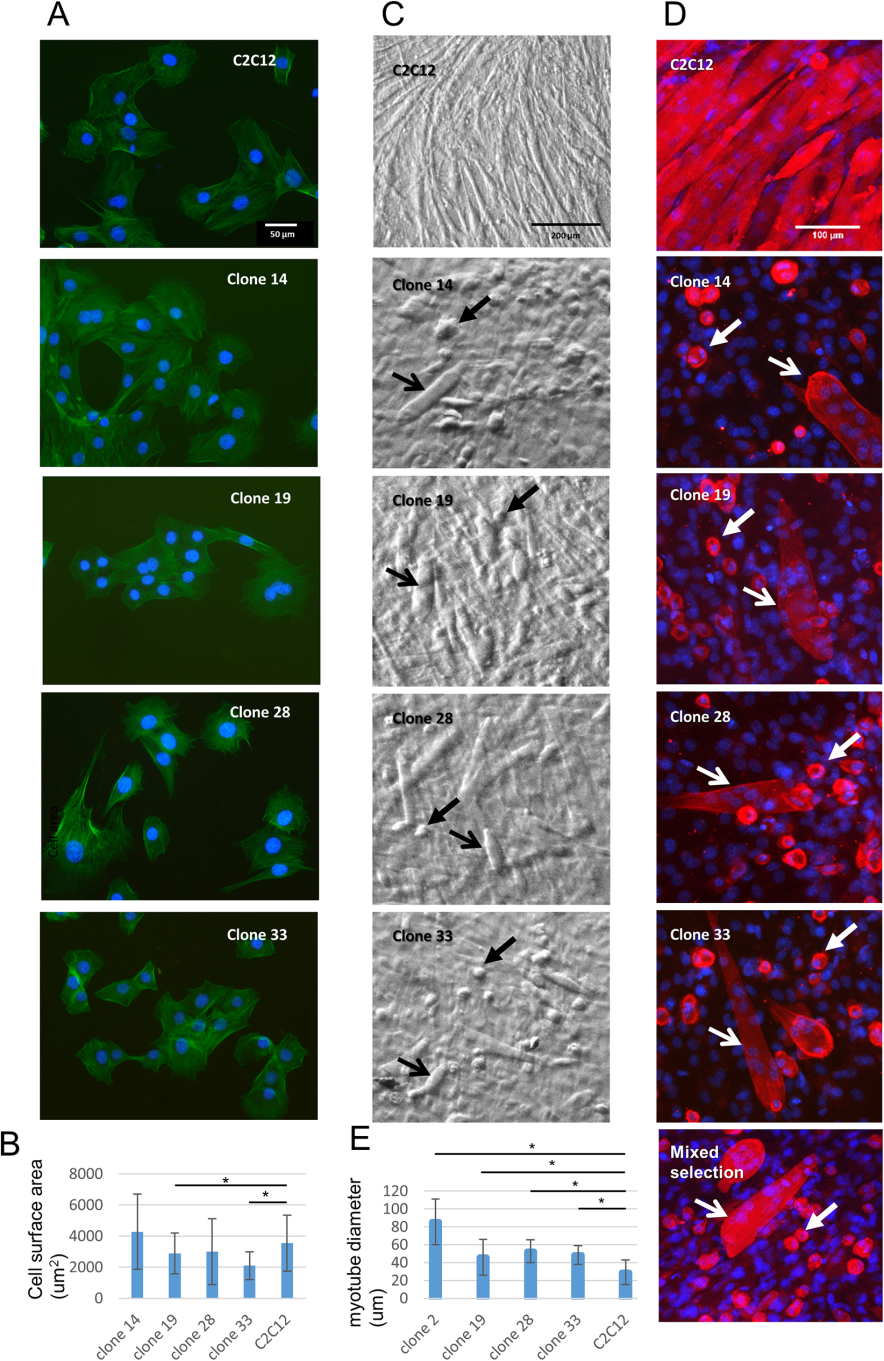
Disruption of *Igfn1* exon 13 in C2C12 cells causes aberrant differentiation patterns. A) Fluorescence images of proliferating clones carrying disruptive mutations on both alleles and stained for phalloidin (green), as labelled. B) Plot of average cell area of proliferating cells for each selected clone, as labelled. Clones 19 and 33 were significantly smaller in size than C2C12 (t-test, *P≤0.05). Error bars represent the standard deviation of each mean. C) Bright field microscopy images of *Igfn1* exon 13 knockout clones and C2C12s after 7 days in differentiation medium. Note prominent round cells (closed arrows) and wide myotubes (open arrows) in all selected clones. D) Fluorescence images of alpha-actinin stained (red) *Igfn1* exon 13 knockout clones after 7 days in differentiation medium. Note the presence of an alpha-actinin-rich ring in single cells (closed white arrows) and wide multinucleated myotubes (open white arrows). E) Plot of average myotube diameter for each selected clone, as labelled. All clones were significantly wider than the C2C12 control (t-test, *P≤0.05).

Clones 14,19, 28 and 33 carrying disruptive mutations were analyzed further. Proliferating cells did not display gross morphological or cytoskeletal differences to the control C2C12 cell line (Fig 7A). The average cell size of clones 19 and 33 was significantly decreased, however other clones did not show a significant size difference compared to C2C12 cells (Fig 7B). Despite this inconsistency in cell size on proliferating medium, all clones showed similar abnormal morphology in DF medium compared to C2C12 cells. After 7 days in DF medium, all clones predominantly formed round cells containing a perinuclear alpha-actinin ring as well as short wide myotubes (Fig 7C and 7D). The shape of round cells and myotubes in brightfield images suggest that they are larger in volume than C2C12 differentiated cells (Fig 7C). To confirm that these abnormal patterns were not a clonal selection artefact, C2C12 cells were transfected with both *Igfn1* CRISPR/Cas9 and homologous recombination donor plasmids and puromycin resistant cells were selected “en masse” and differentiated for 7 days. The results in this mixed population showed the same short wide myotubes and perinuclear alpha-actinin ring phenotypes (Fig 7D, see “Mixed selection” panel). A comparison of the average diameters of differentiated myotubes between KO cell lines and C2C12 control showed a significant increase in myotube width in all IGFN1 KO clones (Fig 7E). It would therefore appear that disruption of *Igfn1* exon 13 leads to fusion defects, aberrant differentiation and increased myotube size.

### IGFN1 KO19 differentiation defects are partially rescued by IGFN1_v1

**Fig 8:**
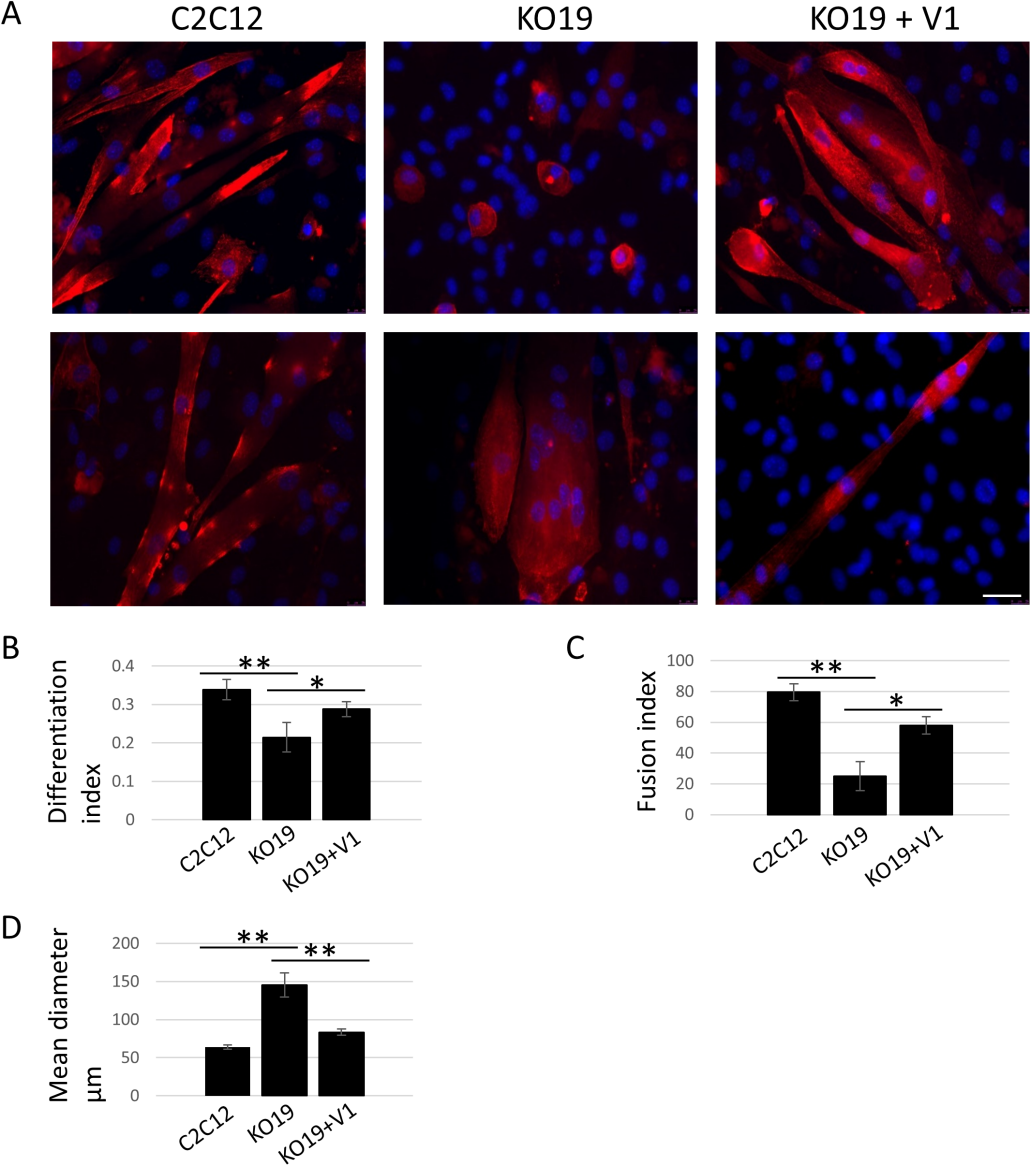
The KO19 cell line displays fusion and differentiation defects partially rescued by expression of IGFN1_v1. A) Representative fluorescence images of myoblasts differentiated for 10 days for C2C12, KO19 and KO19 transfected with IGFN1_v1 coding plasmid (KO19+V1). Cells were stained for alpha-actinin (red) and DAPI (blue). Two examples per cell line shown, as labelled. Scale bar represents 50μm. B) Graph showing the differentiation index for the indicated cell lines, calculated as the proportion of nuclei within an alpha-actinin expressing cell to the total number of nuclei within the same field. Note that KO19 cells have a significantly lower differentiation index than wildtype and rescued cells. C) Fusion index calculated as the percentage of alpha-actinin positive cells with three or more nuclei. KO19 cells have significantly lower fusion index than wildtype and rescued cells. D) Mean diameter of alpha-actinin positive cells containing three or more nuclei, average diameter is significantly higher in knockout cells compared to both the wildtype and the rescue. (**) p<0.01, (*) p<0.05.

Given that all clones with disruptive exon 13 mutations on both alleles displayed similar phenotypes, we selected clone 19 (hereafter referred to as KO19) for further characterizations. To obtain a more accurate picture of the differentiation defects, the size of multinucleated structures, the overall differentiation index and the fusion index were quantified. To compare the size of multinucleated structures the diameter of cells containing three or more nuclei was measured in images from C2C12 and KO19 cells (Fig 8A). A value of three or more nuclei was chosen to distinguish between myotubes originated from multiple fusion events from cells undergoing division. KO19 multinucleated cells have a significantly larger mean diameter than C2C12 myotubes (Fig 8D), indicating that disruption of *Igfn1* exon 13 is causing loss of cell size control in differentiating cells. The differentiation index was quantified by calculating the proportion of nuclei within an alpha-actinin positive structure to the total number of nuclei in a given field. This value was significantly lower in the KO19 cell line (Fig 8B), indicating a delay in expression of the differentiation programme. This was also the case when measuring the fusion index, expressed as the percentage of alpha-actinin positive cells with three or more nuclei to the total number of cells expressing alpha-actinin. The data showed a significantly lower fusion index in KO19 cell line (Fig 8C). Thus, KO19 shows reduced fusion, reduced differentiation and larger multinucleated structures.

In order to test whether the above phenotypes are caused by disruption of *Igfn1* exon 13, and not the result of an off target genetic disruption, a construct coding for recombinant IGFN1_v1:tdTomato (pDEST47_IGFN1_v1_tdTomato) was transfected into the KO19 cell line. Transfected cells were selected by G418 treatment and the mixed resulting population was evaluated for size of multinucleated structures, differentiation index and fusion index, as above. The overall morphology of these cells appeared more similar to that of the C2C12s (Fig 8A) and quantifications demonstrated partial but significant rescue of fusion and differentiation defects in the KO19 transfected cells (Fig 8B–8D). We therefore conclude that IGFN1_v1 plays a critical role in myoblast fusion and differentiation in vitro.

### In vivo overexpression of IGFN1_v1

**Fig 9:**
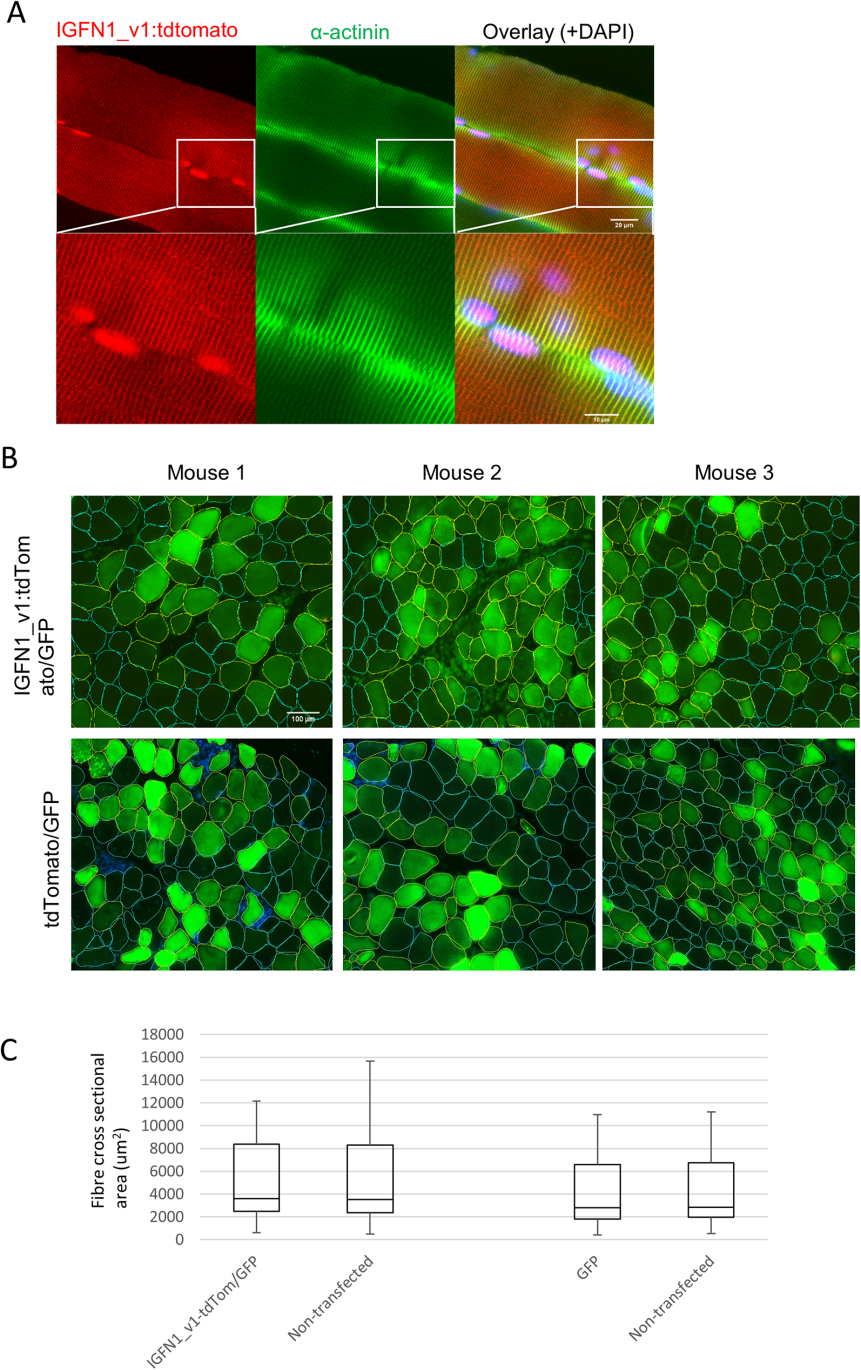
Overexpression of IGFN1_V1:tdTomato in mouse skeletal muscle. (A) Confocal image of a whole mount longitudinal view of mouse EDL/TA muscle fibres electroporated with IGFN1-tdTomato. IGFN1_v1-tdtomato (red) shows nuclear expression and colocalization with alpha-actinin (green) at the Z-disc. The insets (white outlined squares) are shown magnified. The overlay includes DAPI (blue). B) Cross sections of electroporated muscles. The panels show fluorescence images of EDL/TA muscle sections from muscles electroporated with either IGFN1_v1:tdTomato or tdTomato from three male siblings, as indicated. Note that pMAXGFP (GFP) was co-electroporated to facilitate identification of positively transfected fibres. Fibres have been manually labelled with yellow (transfected) or blue (untransfected) outlines for area measurement purposes. (C) Box and whisker plots showing the upper and lower quartiles of cross sectional area measurements for IGFN1_v1:tdTomato+pMAXGFP transfected (IGFN1_v1-tdTom/GFP) and non-transfected fibres and for pMAXGFP transfected (GFP) and non-transfected fibres. The data was pulled from the three electroporated mice illustrated in B. Horizontal and vertical bars represent the median and range of values, respectively. Mann-Whitney U test showed no significant difference between transfected and non-transfected fibres.

The results above and previous expression data [10,11] suggest that IGFN1_v1 plays a role in the regulation of fibre size. To test this, pDEST47_IGFN1_v1_tdTomato was electroporated into adult mouse muscle (5–8 weeks old male C3H/HeJ mice). Electroporations into the TA/EDL muscles showed that recombinant IGFN1_v1 fused to tdTomato retains identical subcellular localizations to those previously reported with IGFN1 fragments and immunofluorescence of adult muscles using anti-IGFN1 antibodies [5]. Longitudinal views of whole mount preparations show both myonuclear and Z-disc localizations (Fig 9A), suggesting that fusion of IGFN1_v1 to the reporter tdTomato had not affected biological function of the former. The effect of IGFN1_v1:tdTomato overexpression was then quantified using electroporated muscles that included a plasmid encoding GFP (pmaxGFP) to facilitate identification of co-electroporated fibres on cross sections. As a control, pmaxGFP alone was electroporated in the contralateral leg. The results from mice showing efficient electroporation (this is, a count of >200 transfected fibres per muscle, Fig 9B, n = 3 mice) show that overexpression of IGFN1_v1:tdTomato does not cause a significant change in the cross sectional area of transfected fibres (Fig 9C). As expected, overexpression of GFP alone does not affect fibre size (Fig 9C).

### In vivo expression of CRISPR/Cas9 vector targeting exon 13

**Fig 10:**
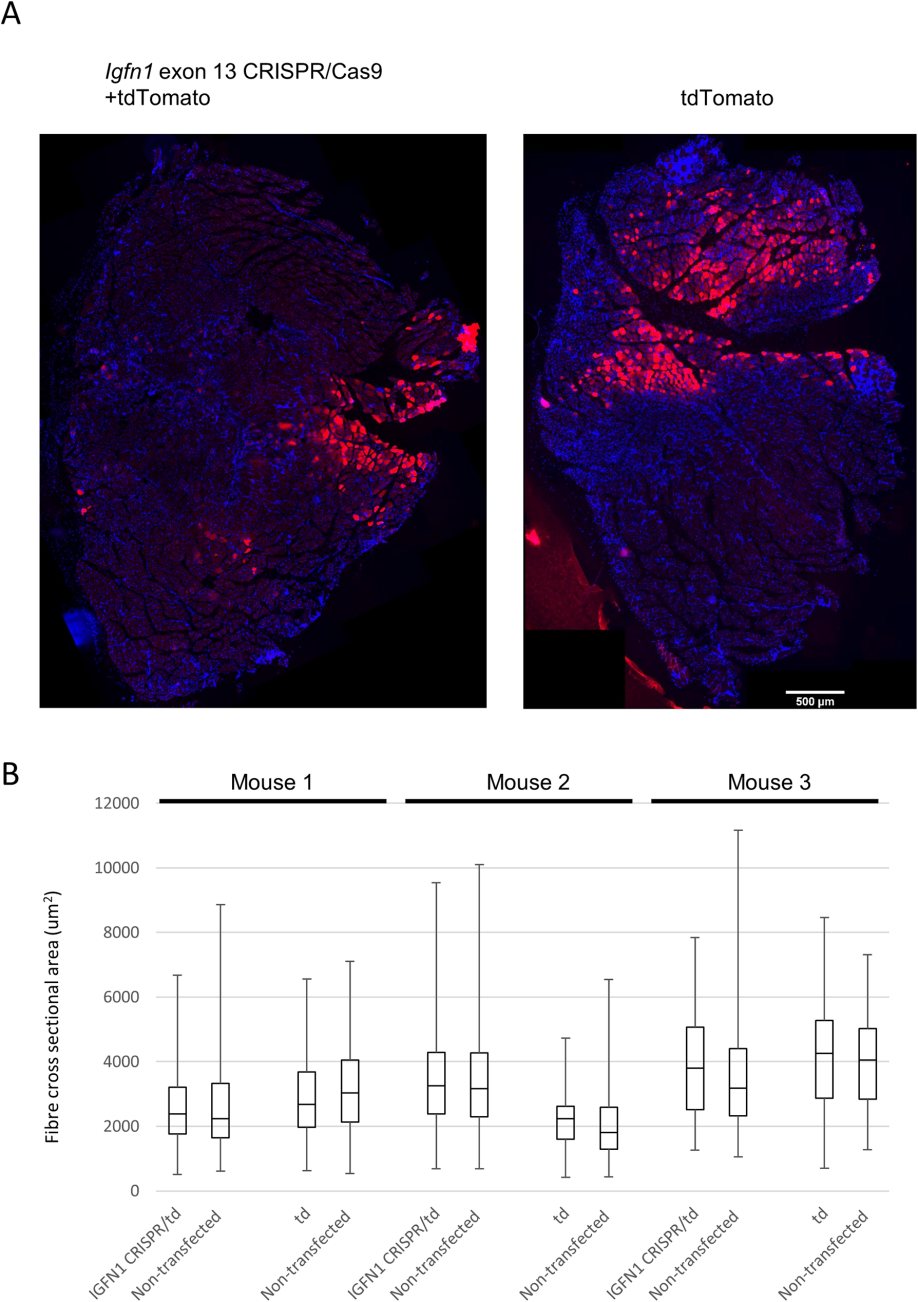
Expression of *Igfn1* exon 13 CRISPR/Cas9 in vivo does not cause significant fibre size change. A) Fluorescence images of cross sections from whole EDL/TA muscles electroporated with *Igfn1* exon 13 CRISPR/Cas9 +tdTomato or tdTomato alone, as indicated. B) Box and whisker plots showing the upper and lower quartiles of cross sectional area measurements for *Igfn1* exon 13 CRISPR/Cas9+tdTomato transfected (IGFN1 CRISPR/td) and non-transfected fibres and for tdTomato (td) transfected and non-transfected fibres. Quantifications were carried out for three mice independently, as indicated. Horizontal and vertical bars represent the median and range of values, respectively. Mann-Whitney U test showed no significant difference between transfected and non-transfected fibres.

The in vitro results showed that deletion of *Igfn1* exon 13 in C2C12 myoblasts caused abnormal differentiation. In an attempt to study the possible relevance of these findings in vivo, the same targeting construct was electroporated in the TA/EDL muscles of 5–8 weeks old C3H/HeJ mice. Transfected fibres were identified by co-electroporating with a vector encoding tdTomato as reporter, at concentration ratios of 1 (tdTomato reporter): 2 (*Igfn1* exon 13 CRISPR/Cas9 vector). Constructs were allowed to express for 21 days or more to facilitate targeting of multiple nuclei within the transfected fibres. Following this long period, fluorescent signal remained strong, indicating that electroporation efficiencies were high (see illustrative example in Fig 10A showing a cross sectional view of the whole TA/EDL muscles) and that plasmids had not been degraded.

Cross sectional area of transfected fibres was measured and compared with untransfected fibres in the same region of the muscle. Quantifications were carried out from three mice without pulling, since the regions within the TA/EDL muscle that were transfected occurred at different locations for each mouse, as assessed by the final stitching of tiled images [19] (Fig 10A). Comparisons between transfected and untransfected fibres from the same region showed that there were no significant fibre size changes in any of the three mice analyzed (Fig 10B). Genomic DNA from the remaining tissue blocks of electroporated mice was purified and amplicons of the *Igfn1* exon 13 targeted region amplified by PCR and Sanger sequenced. This was attempted to test if traces of the sequencing results showed a drop in the quality of the sequence from the targeted site as a result of presence of mutations in the mixed amplicon. The results showed a very small drop in sequence quality from the targeted site (data not shown), indicating that the overwhelming presence of untransfected fibres and non argeted alleles probably prevents detection of mutations by this method. Consistent with this, T7EN digestion of the amplicon also failed to detect digested products Next generation sequencing was not undertaken.

## Discussion

*Igfn1* is a large and complex locus and therefore challenging to analyze. Here, we attempted to assign an overall role to the major isoforms using the myoblast C2C12 cell line, as this is a well-established cellular model of in vitro muscle differentiation. In our hands, C2C12 cells from different sources or passages were often inconsistent in the paucity of differentiation. To minimize this potentially confounding effect, elimination of IGFN1 isoforms was attempted by different shRNAs and individual clones as well as mass selection was undertaken. Partial but evident knock-down of putative isoforms including IGFN1_v1 was obtained in several independently generated cell lines. These cell lines showed a total lack of fusion. Remarkably, the fusion defect did not prevent expression of alpha-actinin and the formation of a primitive sarcomeric-like pattern in single cells, suggesting that myoblast fusion and differentiation respond to independent regulatory cues. The fusion defect was not rescued by full length IGFN1 or IGFN1_v1 in transiently transfected cells or stably selected cell lines (data not shown), suggesting that neither IGFN1 or IGFN1_v1 are sufficient for myoblast fusion. Therefore, the possibility of fusion defects in the various knock-down cell lines being the result of tissue culture artefacts could not be strictly ruled out. A more direct strategy was then adopted by generating a number of C2C12 derived clones carrying deletions or mutations in *Igfn1* exon 13, which encodes most of the second globular domain. It was anticipated that this deletion will not eliminate all isoforms, but those containing the full set of N-terminal globular domains. The differentiation patterns observed in these cell lines were remarkably consistent, producing mainly singly nucleated actin rich cells but also multinucleated structures that appeared more voluminous.

The fusion defect and size of myotubes was quantified in KO19, a cell line with a homozygous deletion of *Igfn1* exon 13. Fusion and differentiation indexes were significantly reduced. However, the myotubes produced were significantly larger. Crucially, these defects were partially rescued by reexpression of IGFN1_v1, indicating that this isoform plays roles in fusion and the regulation of myotube size. The potential inhibitory effect of IGFN1_v1 on myotube size suggested by these results would be consistent with the reported levels of expression of *Igfn1* sharply increasing in atrophy promoting conditions, such as denervation [11] or upregulation of myostatin signaling [10]. Thus, a possible interpretation is that IGFN1_v1 is required to negatively regulate myotube size in differentiated C2C12 cells, therefore lack of IGFN1_v1 in the KO19 cell line results in larger cells. However, a role for IGFN1_v1 in fibre size was not confirmed in our in vivo experiments, as overexpression of IGFN1_v1 in electroporated muscles did not result in fibre size changes. It is plausible that for IGFN1_v1 (or other IGFN1 isoforms) to have a cooperative role in promoting atrophy in vivo, an active signaling pathway mediating atrophy must be already in place. Since mice that were electroporated with IGFN1_v1 coding constructs were not challenged by denervation, a potential synergistic role of IGFN1_v1 to the atrophy pathway was not detected in our quantifications. The results indicate that overexpression of IGFN1_v1 is not sufficient to cause atrophy in vivo. Given the modular domain composition of all IGFN1 isoforms, it is likely that any contribution to atrophy relies on scaffolding roles, bringing together signaling factors to relevant subcellular localizations.

We then attempted to determine whether expression levels of IGFN1/IGFN1_v1 at rest are critical for muscle fibre maintenance. For this, the highly efficient CRISPR/Cas9 construct targeting exon 13 was electroporated and allowed to express for at least three weeks. The assumption given the variety of mutations identified in vitro was that the CRISPR/Cas9 construct targeting *Igfn1* exon 13 would also generate mutations in this exon in vivo. There were no differences in mean fibre size between transfected and non-transfected fibres from the same muscle region. The lack of observed effects with the mutagenic CRISPR/Cas9 vector or the IGFN1_v1 overexpression experiments above was not caused by electroporation artefacts or lack of power in the analyses, as we have consistently observed a significant increase of fibre size with other plasmids using the same experimental design (e.g., myostatin TALENS or plasmid encoding ZAK, unpublished observations). Sanger sequencing did not prove to be a suitable method to detect mutations in genomic preparations from tissues that would contain a minority of targeted alleles. Consequently, the actual efficiency of the targeting vector in vivo remains to be determined. In conclusion, our results indicate a role for IGFN1_v1 in myoblast in vitro fusion and differentiation. In vivo models need to be developed to fully test the role of IGFN1 isoforms in muscle maintenance or development.

## Materials and methods

### Targeting and expression constructs

A short hairpin RNA (shRNA) was selected against the common 3’UTR sequence [5]. The targeted sequence is GCTCAGAGTGACCCTGTTACT. The oligos used to generate the sh1-IGFN1 shRNA were as follows: Forward: CACCGC TCAGAG TGACCC TGTTAC TCGAAA GTAACA GGGTCA CTCTGA GC and Reverse: AAAAGC TCAG A GTGACC CTGTTA CTTTCG AGTAAC AGGGTC ACTCTG AGC. Forward and reverse oligos were annealed and ligated to the pENTR/U6 entry vector according to the manufacturer’s protocol (BLOCK-iT U6 RNAi Entry Vector Kit, Invitrogen) to yield pENTR/U6*I*sh-1-IGFN1. A negative control construct, pENTR/U6*LacZ*, was also produced as described in the manufacturer’s instructions. C2C12 cells (ATCC catalogue number: CRL-1772TM) were seeded on 100-mm tissue culture dishes, and transfected using FuGENE HD (Roche) following manufacturer’s instructions with pENTR/U6*I*sh-1-IGFN1 in combination with a neomycin resistance gene containing vector to enable selection. After 24h, growth medium was replaced with selection medium containing 600μg/ml G418. The selection medium was changed every day for two weeks until single colonies could be isolated. Single colonies were picked, expanded and induced to differentiate when they reached 90–100% confluence. Cells were collected and samples processed for western blots. Presence of pENTR/U6*I*sh-1-IGFN1 was confirmed by PCR (data not shown).

To express the shRNA constructs transiently in C2C12 cells the shRNA constructs from the pENTR/U6*I*sh-1-IGFN1 and the negative control pENTR/U6*LacZ* entry vectors were shuttled into the Gateway pAd/BLOCK-iT™-DEST destination vector following manufacturer’s instructions (BLOCK-iT™ Adenoviral RNAi Expression System, Invitrogen) to generate pAd/BLOCK-iT™-sh-1-IGFN1 and pAd/BLOCK-iT™-*LacZ*, respectively. Viruses were generated as per manufacturer’s instructions (BLOCK-iT™ Adenoviral RNAi Expression System, Invitrogen). C2C12 cells were transduced with either pAd/BLOCK-iT™-sh-1-IGFN1 or pAd/BLOCK-iT™-*LacZ* at D0, D2 or D4 of differentiation as per manufacturer's’ instructions (Invitrogen) and then left to differentiate.

In addition, a lentivirus clone (SHVRSC-TRCN0000109575, MISSION® shRNA Lentiviral technology, SIGMA) targeting a different portion of the 3’UTR of *Igfn1* and referred in the text as sh-2-IGFN1 was selected for testing using as negative control the MISSION® Non-target shRNA control transduction particles (SHC002V, SIGMA). The targeted sequence was CCCTACAGCTATGAGATGGAT. MISSION® shRNA lentiviral transduction particles against the *Igfn1* 3’UTR (CCGGCC CTACAG CTATGA GATGGA TCTCGA GATCCA TCTCAT AGCTGT AGGGTT TTTG), and shRNA control transduction particles were used. Lentiviral particles were transduced into C2C12 myoblasts following manufacturer’s recommendations to generate stable cell-lines positive for the shRNA (SIGMA-ALDRICH). Two cell lines, designated clones Igfn1KD2 and Igfn1KD3 showed down-regulation of IGFN1 isoforms on Western blots.

To generate a strong alternative reporter the cycle 3 GFP was replaced by tdTomato in the vector (Life Technologies). For this, tdTomato was amplified (primers: tdTomato-forward: ATC TAG AAT GGC TAG CGT GAG CAA GGG CGA GGA G; tdTomato-reverse: CGT TGG GAT CTT TCG AAT TAC TTG TAC AGC TCG TCC ATG C) from tdTomato from pRSET-B_tdTomato (a kind gift of Dr. Paul Pryor) with modified primers that include NheI and BstBI restriction sites and recloned into NheI/BstBI digested pcDNA™-DEST47 to generate the destination vector pcDNA-DEST47-tdTomato. This destination vector was then used to generate IGFN1 protein variants fused to tdTomato (pDEST47_IGFN1_tdTomato and pDEST47_IGFN1_v1_tdTomato) using the Gateway cloning protocol following manufacturer's instructions (Life Technologies). pDEST47_tdTomato and pmaxGFP (amaxa Inc., USA) were used as reporter vectors used to facilitate identification of transfected fibres in co-electroporation experiments, as for quantifications purposes fluorescence from IGFN1_v1_tdTomato fusion protein was not strong enough. To generate pDEST47_tdTomato a small DNA fragment (ACGTGTCCGAGCGCATTGACC) was cloned into pENTR-D-TOPO and used in an L/R gateway reaction with pcDNA-DEST47-tdTomato.

A CRISPR/Cas9 vector containing a sgRNA targeting *Igfn1* exon 13 was generated (MCP10032-CG01-02-C, GeneCopoeia) to work in combination with the plasmid containing homologous recombination sequences as indicated in Fig 5B (DOR-MCP10032-02, GeneCopoeia).

### Measurement of apoptosis in the Igfn1KD1 cell line

The level of active caspases were assessed using the CHEMICON®’s CaspaTag™ *In Situ* Caspase Detection Kit. The assay was performed on control and Igfn1KD1 cell lines at D4 of differentiation. Confocal microscopy was then used to determine the amount of fluorescence, which is a direct measure of the number of active caspase positive cells. To quantify the number of cells undergoing apoptosis in the control and Igfn1KD1 cell lines, pictures were taken from six independent experiments and, in an un-biased fashion, the number of apoptotic cells and number of non-apoptotic cells were counted. The ratio of apoptotic over non-apoptotic cells was then calculated for each experiment. Comparisons of the number of cells undergoing apoptosis from the six experiments were then made between control and *Igfn1* knock-down cells using a student’s t-test.

### Rac1 activation assay

To measure the levels of active Rac1 protein the Rac1 activation assay Biochem kit™ from Cytoskeleton was employed. Protein was extracted from the control and Igfn1KD1 cell lines at day 2 of differentiation, and incubated with the GST-tagged PAK-PBD agarose beads to “pull-down” active Rac1-GTP from the lysates. Pulled-down active Rac1 was then detected on western blots using an anti-Rac1 antibody. A small sample of the protein lysate before addition of the GST-tagged PAK-PBD beads was also run on western blots to determine total Rac1 protein content in the lysate. Results were obtained from two independent experiments.

### Antibodies

Previously generated rabbit polyclonal antibodies were used this work. ab-US42 (epitope: CSTPDFK QKPVTL ALPEGKN), ab-US43 (epitope: SVSLKD SGEYKA VAKN), ab-kip2a (epitope: FQSRLP VQAAWR KDGNEV) and ab-kip2b (epitope: VKSPTY QDPDLS QKPRFL). Antibodies were affinity purified and recovered at the following concentrations: ab-US42 (2.2mg/ml; dilution for WB: 1:500), ab-US43 (1.35mg/ml; dilution for WB: 1:500), ab-kip2a (1.14mg/ml; dilution for WB: 1:3000) and ab-kip2b (0.6mg/ml; dilution for WB: 1:500). Additional antibodies used were: RR90 (against FLNC, kind gift of Peter van der Ven [20]), myogenin (Abcam), EA-53 (against alpha-actinin, SIGMA), GAPDH (Abcam), rabbit polyclonal anti-V5 (SIGMA), goat anti rabbit IgG TRITC-conjugated (SIGMA), goat anti rabbit IgG FITC-conjugated (SIGMA), goat anti mouse IgG FITC-conjugated (SIGMA), goat anti mouse IgA FITC-conjugated (SIGMA) and goat anti mouse IgG TRITC-conjugated (SIGMA). Dilutions were as indicated or recommended by the manufacturer.

### Sample preparation

C2C12 cells were cultured in Dulbecco’s modified Eagle’s medium (DMEM) supplemented with 10% fetal bovine serum (FBS) and 1% penicillin/streptomycin. C2C12 cells were induced to differentiate by switching to DMEM containing 2% horse serum and 1% penicillin/streptomycin. For Western blots, cells were collected by washing twice in ice-cold phosphate-buffered saline and then lysed for 30 minutes at 4^°^C with 500μl of NP-40 lysis buffer containing 50mM Tris-HCl pH8, 150mM NaCl, 1%NP40 and protease inhibitors (Roche). Lysates were then subjected to three freeze/thaw cycles and clarified by centrifugation at 14,000g for 10 minutes at 4^°^C. Protein concentrations were determined by the Bradford reagent (SIGMA). Routinely, 15μg of protein from C2C12 cell lysates were loaded per protein gel well.

### Mismatch cleavage assay

The targeting efficiency of the IGFN1 CRISPR/Cas9 vector (GeneCopoeia Inc) was tested using a mismatch cleavage assay. 3T3 cells (ATCC catalogue number: CRL-1658) were grown in a T25 flask to ~90% confluency before transfection. 12μg of IGFN1 CRISPR/Cas9 plasmid was transfected into 3T3 cells using GenJet in vitro DNA transfection reagent (SignaGen Laboratories). Cells were harvested after 48 hours. Genomic DNA was extracted from transfected 3T3 cells using GenElute™ Mammalian Genomic DNA Miniprep Kit (SIGMA). The IGFN1 CRISPR/Cas9 targeted region was amplified by PCR using the following primers: Arm_L: CGGGCAGCTGATAAGTGTTA, Arm_R: TGTGGGCAGAGGCATGTCATA. The PCR products were subjected to DNA re-hybridization by incubation with 10X NEB buffer 2 at 95°C for 10 minutes, then the incubation temperature was slowly dropped until 25°C with ramp rate of -1°C/minute. The hybrid PCR product was incubated with T7 endonuclease I at 37°C for 2–3 hours. The result was checked on a 2% (w/vol) agarose gel.

### Establishment of cell lines with CRISPR/Cas9 mediated disruptive mutations in *Igfn1* exon 13

C2C2 cells were co-transfected with the *Igfn1* exon 13 CRISPR/Cas 9 targeting vectors described above using GenJet In Vitro DNA Transfection Reagent for C2C12 Cells (SignaGen Laboratories). 48 hours after transfection, puromycin (4μg/ml) was added into the growth medium to select for colonies expressing the homologous recombination cassette for 7 days. Clonal selection by serial dilutions into 96 well plates was undertaken to generate colonies from single cells. To test for homologous recombination, a forward primers were placed on the *Igfn1* genomic sequence before homologous recombination left arm (BF_L: TGC TCA TGT GCC TAG CAT GT) to be used in combination with a reverse primer within the GFP/Puromycin cassette (HR_L: CAG ATC GTA CCA AGG GCG AA) and a reverse primer was placed after homologous recombination right arm (AF_R: CTA AGA GGT GTT CCA CTC CCA A) to be used with a forward primer within the GFP/Puromycin cassette (HR_R: AGG AAC GAA GTC CCT AAA GAA ACA). To test for mutations generated by NHEJ, forward and reverse PCR primers were located on the left and right homologous recombination arms, respectively (Arm_L: CGG GCA GCT GAT AAG TGT TA; Arm_R: TGT GGG CAG AGG CAT GTC ATA). See Fig 6A for an schematic of primer locations.

### qRT-PCR assays on *Igfn1* KO C2C12 derived cell lines

To detect the expression of endogenous *Igfn1* in C2C12 and *Igfn1* KO cell lines, mRNA was extracted from C2C12 myoblasts using GenElute DIRECT mRNA MINIPREP KIT (SIGMA), and the cDNA was converted from mRNA by Reverse transcriptase (QIAGEN). The cDNA concentration of each sample was measured, diluted to the same concentration and mixed with primers and SYBR Green Master Mix (Applied Biosystems) to form RT-PCR reactions on an optical 96 well plate. The reactions for *Igfn1* and housekeeping reference gene were carried out using the following primer sets: RT-PCR IGFN1-Forward: GGT ATC GTC GAC TTC CGGG; RT-PCR IGFN1-Reverse: GTC AAA CGT AGC GAC CCC T; RT-PCR HPRT-Forward: GTT GGA TACA GGC CAG ACT TTG TTG; RT-PCR HPRT-Reverse: GAT TCA ACT TGC GCT CAT CTT AGG C.

### Immunofluorescence

For immunofluorescence analysis on C2C12 myoblasts or myotubes, cells were grown or differentiated to the required time points and then fixed with 4% PFA.

To label actin in C2C12 cells, fixed cells were incubated with Alexa Fluor 488® phalloidin (Life Technologies, 1:100 dilution) or Phalloidin-TRITC (P1951, SIGMA, 1:100 dilution) for 30 minutes then washed twice with PBS.

To label alpha-actin in the cells (antibody information on section 2.8.6), fixed cells were treated with with 0.5% Triton X-100 in PBS for 1 minute to permeabilize the cells, then blocked in 4% bovine serum albumin (BSA,w/v)/PBS for 30 minutes, then incubated with Anti-Sarcomeric Alpha Actinin antibody [EA-53] (abcam, 1:150 dilution in 4% BSA/PBS) overnight at 4°C. The next day, cells were washed three times with PBS (5–10 minutes per wash), then incubated with FITC conjugated secondary antibody (secondary information in section 2.8.7) in 4% BSA for 1 hour in darkness, afterwards, the cells were washed three times in PBS (5–10 minutes per wash). Cells were mounted on slides with either vectashield (Vector Laboratories, USA) or MOWIOL (48g/l MOWIOL [CALBIOCHEM], 10% glycerol, 50mM Tris pH8.5), with added DAPI. Fluorescence images were captured with a Microphot-FX Research Microscope (Nikon).

Cell lines C2C12 (parental control) KO19 (deleted for *Igfn1* exon 13) and KO19+IGFN1_V1 (stable KO19 transfected with IGFN1_v1) (see main text for details) cells were grown to confluency on collagen coated 6-well plates in growth medium (DMEM 10% FBS) before being switched to differentiation medium (DMEM 2%FBS) which was replaced daily for 10 days. Cells were then fixed with 1:1 acetone:methanol, washed in PBS, permeabilized in PBS 0.1% triton 5mins, blocked in 4% BSA 1hour, incubated overnight in EA53 (1:150), washed and incubated in anti-mouse IgG TRITC for 1 hour, washed and coverslips mounted using MOWIOL. Differentiation and fusion indexes calculated from collected images obtained with the same settings as explained in the main text. The mean largest diameter of alpha-actinin expressing cells (with >3 nuclei) was taken from the widest point of the cell using a line at 90 degrees to the longest axis. A minimum of 50 alpha-actinin expressing cells were recorded for each cell line.

### In vivo electroporation

One hour before the electroporation procedure, mice (C3H/HeJ, 5–8 weeks old) were injected with 10μl of 0.4U/μl hyaluronidase (in 0.9% saline). DNA was diluted to 800–1200 ng/μl in ddH_2_O, then 10μl of DNA samples were loaded into a sterile syringe. Mice were placed in an anesthetizing box with 4% isoflurane in O_2_ supplied by an approved gas anaesthetic machine until deeply anaesthetised. Mice were then removed to a heating pad (37°C) and continually anesthetized with a rodent face mask. Toe pinch reflex was used to test the anaesthetic depth. The extensor digitorum longus/ tibialis anterior (EDL/TA) muscles were chosen for these experiments as they are physically confined within the hindlimb and easy to access. Three mm wide electrodes were placed within the muscle and DNA injected between the electrode sites. Pulses were delivered using a NEPA21 machine (Nepagene, Japan). Three 50-msec-long pulses at 100V followed by three more pulses of the opposite polarity were administered to each injection site at a rate of one pulse per sec. Mice were sacrificed at 7 to 30 days after the electroporation. EDL/TA muscles were dissected, fixed for 10 minutes in 4% PFA and snap frozen in liquid nitrogen-cooled isopentane and stored at -80°C. 12 micron thick cross sections were produced for quantifications of fibre size using a cryostat. Animals were sacrificed by a schedule one killing (cervical dislocation). All animal procedures have been carried with approval from the University of York Ethics committee and followed the UK Animals (Scientific Procedures) Act 1986 Amendment Regulations 2012, performed by under project licence PPL 70/6827 within an approved establishment (licence 5002510).

### Statistics

Tests were performed using R (https://www.r-project.org/). t-tests were performed after normality tests using D’ Agostino’s test. Data which did not pass the normality test was log-transformed and re-tested for normality. Normally distributed data was analysed using either paired or un-paired t-tests. Non-normally distributed data were analysed using either Wilcoxon signed-rank tests (paired data) or Mann-Whitney tests (unpaired data). To analyse the difference among three or more samples, single factor ANOVA was used in normally distributed data, and Kruskal-Wallis H testing was used in non-normally distributed data. Single factor ANOVA tests were followed by post hoc tests to analyse the differences between samples, in which Tukey's honestly significant difference (HSD) post hoc test was used to analyse data containing homogeneity of variances, whereas the Games Howell post hoc test was used to analyse data not containing homogeneity of variances.

## References

[1] Beatham J, Romero R, Townsend SKM, Hacker T, van der Ven PFM, Blanco G. Filamin C interacts with the muscular dystrophy KY protein and is abnormally distributed in mouse KY deficient muscle fibres. Hum Mol Genet. 2004;13: 2863–2874. doi: 10.1093/hmg/ddh308 15385448 10.1093/hmg/ddh308

[2] Blanco G, Coulton GR, Biggin A, Grainge C, Moss J, Barrett M, et al The kyphoscoliosis (ky) mouse is deficient in hypertrophic responses and is caused by a mutation in a novel muscle-specific protein. Hum Mol Genet. 2001;10: 9–16. 11136708 10.1093/hmg/10.1.9

[3] Hedberg-Oldfors C, Darin N, Olsson Engman M, Orfanos Z, Thomsen C, van der Ven PFM, et al A new early-onset neuromuscular disorder associated with kyphoscoliosis peptidase (KY) deficiency. Eur J Hum Genet. 2016;24: 1771–1777. doi: 10.1038/ejhg.2016.98 27485408 10.1038/ejhg.2016.98 PMC5117942

[4] Straussberg R, Schottmann G, Sadeh M, Gill E, Seifert F, Halevy A, et al Kyphoscoliosis peptidase (KY) mutation causes a novel congenital myopathy with core targetoid defects. Acta Neuropathol. 2016;132: 475–478. doi: 10.1007/s00401-016-1602-9 27484770 10.1007/s00401-016-1602-9

[5] Baker J, Riley G, Romero MR, Haynes AR, Hilton H, Simon M, et al Identification of a Z-band associated protein complex involving KY, FLNC and IGFN1. Exp Cell Res. 2010;316: 1856–1870. doi: 10.1016/j.yexcr.2010.02.027 20206623 10.1016/j.yexcr.2010.02.027

[6] Otey CA, Dixon R, Stack C, Goicoechea SM. Cytoplasmic Ig-domain proteins: Cytoskeletal regulators with a role in human disease. Cell Motil Cytoskeleton. 2009;66: 618–634. doi: 10.1002/cm.20385 19466753 10.1002/cm.20385 PMC2735333

[7] Pinotsis N, Chatziefthimiou SD, Berkemeier F, Beuron F, Mavridis IM, Konarev PV, et al Superhelical Architecture of the Myosin Filament-Linking Protein Myomesin with Unusual Elastic Properties. PLoS Biol. 2012;10: e1001261 doi: 10.1371/journal.pbio.1001261 22347812 10.1371/journal.pbio.1001261 PMC3279516

[8] McPherron AC, Lee SJ. Double muscling in cattle due to mutations in the myostatin gene. Proc Natl Acad Sci U S A. 1997;94: 12457–12461. 9356471 10.1073/pnas.94.23.12457 PMC24998

[9] Rahimov F, King OD, Warsing LC, Powell RE, Emerson CP Jr, Kunkel LM, et al Gene expression profiling of skeletal muscles treated with a soluble activin type IIB receptor. Physiol Genomics. 2011;43: 398–407. doi: 10.1152/physiolgenomics.00223.2010 21266502 10.1152/physiolgenomics.00223.2010 PMC3092338

[10] Chen JL, Walton KL, Winbanks CE, Murphy KT, Thomson RE, Makanji Y, et al Elevated expression of activins promotes muscle wasting and cachexia. FASEB J. 2014;28: 1711–1723. doi: 10.1096/fj.13-245894 24378873 10.1096/fj.13-245894

[11] Mansilla F, Dominguez CAG, Yeadon JE, Corydon TJ, Burden SJ, Knudsen CR. Translation elongation factor eEF1A binds to a novel myosin binding protein-C-like protein. J Cell Biochem. 2008;105: 847–858. doi: 10.1002/jcb.21880 18756455 10.1002/jcb.21880 PMC2597023

[12] Hasty P, Bradley A, Morris JH, Edmondson DG, Venuti JM, Olson EN, et al Muscle deficiency and neonatal death in mice with a targeted mutation in the myogenin gene. Nature. 1993;364: 501–506. doi: 10.1038/364501a0 8393145 10.1038/364501a0

[13] Sanger JW, Chowrashi P, Shaner NC, Spalthoff S, Wang J, Freeman NL, et al Myofibrillogenesis in skeletal muscle cells. Clin Orthop Relat Res. 2002; S153–62. 12394464 10.1097/00003086-200210001-00018

[14] Vasyutina E, Martarelli B, Brakebusch C, Wende H, Birchmeier C. The small G-proteins Rac1 and Cdc42 are essential for myoblast fusion in the mouse. Proc Natl Acad Sci U S A. 2009;106: 8935–8940. doi: 10.1073/pnas.0902501106 19443691 10.1073/pnas.0902501106 PMC2682539

[15] Luo L, Liao YJ, Jan LY, Jan YN. Distinct morphogenetic functions of similar small GTPases: Drosophila Drac1 is involved in axonal outgrowth and myoblast fusion. Genes Dev. 1994;8: 1787–1802. 7958857 10.1101/gad.8.15.1787

[16] Hakeda-Suzuki S, Ng J, Tzu J, Dietzl G, Sun Y, Harms M, et al Rac function and regulation during Drosophila development. Nature. 2002;416: 438–442. doi: 10.1038/416438a 11919634 10.1038/416438a

[17] Fernandes JJ, Atreya KB, Desai KM, Hall RE, Patel MD, Desai AA, et al A dominant negative form of Rac1 affects myogenesis of adult thoracic muscles in Drosophila. Dev Biol. 2005;285: 11–27. doi: 10.1016/j.ydbio.2005.05.040 16125691 10.1016/j.ydbio.2005.05.040

[18] Charrasse S, Comunale F, Fortier M, Portales-Casamar E, Debant A, Gauthier-Rouvière C. M-cadherin activates Rac1 GTPase through the Rho-GEF trio during myoblast fusion. Mol Biol Cell. 2007;18: 1734–1743. doi: 10.1091/mbc.E06-08-0766 17332503 10.1091/mbc.E06-08-0766 PMC1855016

[19] Preibisch S, Saalfeld S, Tomancak P. Globally optimal stitching of tiled 3D microscopic image acquisitions. Bioinformatics. 2009;25: 1463–1465. doi: 10.1093/bioinformatics/btp184 19346324 10.1093/bioinformatics/btp184 PMC2682522

[20] van der Ven PFM, Obermann WMJ, Lemke B, Gautel M, Weber K, Furst DO. Characterization of muscle filamin isoforms suggests a possible role of gamma-filamin/ABP-L in sarcomeric Z-disc formation. Cell Motil Cytoskeleton. 2000;45: 149–162. doi: 10.1002/(SICI)1097-0169(200002)45:2<149::AID-CM6>3.0.CO;2-G 10658210 10.1002/(SICI)1097-0169(200002)45:2<149::AID-CM6>3.0.CO;2-G

